
JOURNAL INFORMATION
==============================
NLM Title Abbreviation: ScientificWorldJournal
Journal ID: ScientificWorldJournal
NLM Journal ID: 101131163
Journal ID: TSWJ

The Scientific World Journal

ISSN: 2356-6140
EISSN: 1537-744X
Publisher: Wiley

ARTICLE INFORMATION
==============================
PMCID: PMC6083934
PMID: 30147550
DOI: 10.1100/tsw.2001.138
Article ID: 283643
Article version: 1
Subjects: Short Report

The Insulin Signaling Pathway and Aging in Drosophila

Partridge Linda
Department of Biology, University College London, Wolfson House, 4 Stephenson Way, London NW1 2HE, UK
Publication date: 2001
Electronic publication date: 2001 May 1
Volume: 1
First page: 76
Last page: 76
Copyright: Copyright © 2001 Linda Partridge.
Copyright year: 2001
License: This is an open access article distributed under the Creative Commons Attribution License, which permits unrestricted use, distribution, and reproduction in any medium, provided the original work is properly cited.
License URL: https://creativecommons.org/licenses/by/3.0/

==============================
Miami *Nature Biotechnology* Short Reports
*TheScientificWorld* (2001) 1(S3), 76SR
ISSN 1532-2246; DOI 10.1100/TSW.2001.138

### THE INSULIN SIGNALING PATHWAY AND AGING IN *DROSOPHILA*

Linda Partridge

Department of Biology, University College London, Wolfson House, 4 Stephenson Way, London NW1 2HE, UK
ucbhlop@ucl.ac.uk

Model organisms such as yeast, the nematode worm *C. elegans* and the fruit fly *Drosophila* are providing useful insights into the aging process. For information gathered from these organisms to be useful medically, it is important that mechanism of aging be conserved during evolution.

In the nematode worm *C. elegans*, mutation of genes encoding components of the insulin/Insulinlike Growth Factor (IGF) signaling pathway extends the lifespan of the adult worm. They also confer resistance to environmental stresses such as heat and oxidation, and are associated with increased levels of antioxidant defenses. These mutations were discovered because they also control entry to an alternative route of development, to a growth-arrested, non-feeding, fat-storing, stress-resistant dauer larva that is produced in response to crowding. Thus, the extension of adult lifespan by these mutations could be a peculiarity of *C. elegans*, resulting from the pathway's function in the regulation of the dauer developmental route.

We have examined the effects on aging of mutations in genes in the homologous pathway in another model organism, *Drosophila*. Mutants have been made in several of the genes in the insulin pathway, to study effects on growth during the pre-adult period. Over-expression of several components of the pathway increases growth rate and final size. We find that a mutation in one of these genes, *chico*, a homologue of the vertebrate insulin receptor substrates 1-4, increases mean and maximum lifespan of the fly. Unlike the findings in *C. elegans*, we find no association between mutations in the pathway and resistance to environmental stress. Nor did we find that elevated antioxidant defences, dwarfism, high lipid content or reduced reproductive rate, all associated with in *chico*, played any role in extension of lifespan. Thus the role of the insulin/IGF signaling pathway in regulating lifespan is evolutionary conserved, but many of the other conserved effects of the pathway seem not to be causal in extension of lifespan.

We tested the hypothesis that insulin/IGF signaling mediates the extension of lifespan by caloric restriction. If insulin/IGF mutants are already somewhat calorically restricted by their genotype, then we would predict that their lifespan would be maximal at a higher food level than would that of controls, and that the mutants would be more susceptible to the effects of starvation. Both of these predictions were confirmed, demonstrating that in *Drosophila* lifespan extension by caloric restriction is mediated by insulin/IGF signaling.

**REFERENCES.**

1. Sgrò, C. and Partridge, L. (1999) Science, 286, 2521-2524
2. Partridge, L., Prowse, N., and Pignatelli, P. (1999) Proc. R. Soc. Lond. B 266, 255-261
3. Rogina, B., Vaupel, J.W., Partridge, L., and Helfand, S.L. (1998) Curr. Biol. 8, 475-478
4. Chapman, T. and Partridge, L. (1996) Proc. R. Soc. Lond. B 263, 755-759
5. Chapman, T., Liddle, L.F., Kalb, J.M., Wolfner, M.F., and Partridge, L. (1995) Nature 373, 241-244
6. Partridge, L. and Barton, N.H. (1993) Nature 362, 305-311
